# Book Review

**Published:** 1925-01

**Authors:** 


### Reviews of Books.

**Rheumatic Heart Disease.** By Carey F. Coombs, M.D., F.R.C.P. Lond. With an introductory chapter by F. J. Poynton, M.D., F.R.C.P. Lond. Pp. xxiii., 376. Bristol: John Wright & Sons, Ltd. 1924. Price 12s. 6d.—This book deals with the cardiac lesions of rheumatism, but the different manifestations, which are usually pigeon-holed into definite named diseases in the current text books, are not unduly stressed; the author shows that these diseases are due to one and the same tissue change, and that the differences are caused by variability of local intensity. Whether rheumatic carditis can be separated from rheumatism is another question, but the separation is justified for the production of a monograph. The ætiology, so far as it is known, is treated carefully, but with reserve. *Streptococci* in general and the *S. rheumaticus* of Poynton and Paine in particular, are regarded as the underlying exciting cause, and this will meet with general agreement. Our lamentable ignorance of this most puzzling group of micro-organisms is noted, and the author points out the promise of labour and reward in the further investigation of this part of the question. The morbid changes in the different parts of the heart are lucidly described and well illustrated by admirable photo-micrographs. The unity of these changes is well demonstrated, and thus goes far to support the main thesis. The chapters on symptomatology, prognosis and treatment will appeal to most readers. This part of the book is of much interest, for the author has not been content to hand on the traditional teaching, but has based his conclusions on the findings of a large number of cases seen and followed carefully by himself for years. This by itself should be sufficient to ensure a critical reading. In addition proof is afforded that some of the current teaching is fallacious, and therefore dangerous ; thus the rarity of much pericardial effusion and the danger of attempting aspiration in rheumatic carditis is an example out of several which will make an appeal to many practitioners. One or two minor points in chapters on prognosis appear to deserve more notice in subsequent editions ; the solution of the question of present infection in a given case might receive help from the finding of an alteration of the Arneth index and later a mononucleosis. The prognostic significance of sinus arrhythmia, first pointed out by Sir James Mackenzie, and that of an embryocardial rhythm are not noted. Changes in the pulse pressure, not widely known, are pointed out, but notes on the alterations of the tone phases of the pulse pressure in an individual case might be helpful. The study of the vital capacity or the duration of voluntary apnœa are also of help, but are not noted. Those methods which give a definite figure, or percentage, so dear to the hearts of the younger generation at least, deserve more notice. A slight alteration of a sound, though full of significance to the practised cardiologist, is not appreciated by the general practitioner. Apart from this the prognosis is ably considered and all contingencies are fully discussed. Opinions on the permissibility of marriage, though they may appear to be somewhat heretical, are supported by prolonged observation of cases and are given with duly expressed reserve. The schemes for a fuller investigation of rheumatism, social questions and means for the prevention of the condition are of manifest importance from a national standpoint, and will appeal to most readers. The book is well turned out, convenient to handle and free from technical faults. It is original, well written and of interest throughout. The illustrations are clear and the index is good. It should be of great help to the practitioner, and its appearance just now, when the importance of rheumatism has permeated the thoughts of the laity, is timed to add to its utility.

**Pneumonia: its Pathology, Diagnosis, Prognosis, and Treatment.** By the late R. Murray Leslie, M.A., B.Sc., M.D. Revised and edited by J. Browning Alexander, M.D., M.R.C.P. Pp. xiv., 351. London : William Heinemann. 1924. Price 12s. 6d. net.—Dr. Alexander is to be congratulated on the skill and judgment with which he has accomplished the work taken in hand by the late Dr. Murray Leslie, whose long and wide experience of pulmonary disease qualified him for the production of such a volume as this, that is to say, a comprehensive study of pneumonia with particular attention to the clinical aspect of the disease. The book is a balanced and critical review of the whole subject, in which both old and new views are brought under consideration. Almost half the pages are devoted to a discussion of the treatment of pneumonia, its varieties and complications. A cautious support is given to the use of vaccines in the earliest days of the disease. There is an interesting account of the researches into the various types of the pneumococcus recently carried out at the Rockefeller Institute, with their bearing on the use of specific sera. It is a pleasant volume to handle, well bound and clearly printed on an attractive paper. The style is easy and the arrangement of chapters and paragraphs well conceived. For these reasons, as well as for its general reliability and comprehensiveness, this book should commend itself to a wide circle of readers.

**Anæmia : its Causes and Modern Treatment.** By ARTHUR W. FULLER, M.D.Edin. Second Edition. Pp. 138. London : H. K. Lewis & Co. Ltd. 1924. Price 6s.—This book deals with the subject of anæmia, and its treatment, mainly in its clinical aspect. All forms of anæmia, both primary and secondary, are included, and the relationship of anæmia to other diseases, such as neurasthenia, is fully discussed. The best chapters are those devoted to treatment, and the author emphasizes the importance of rest, exercise, air and light, in this condition, and more particularly the treatment by deep subcutaneous injections of iron and arsenic in isotonic serum. The gradual disappearance of chlorosis during recent years has been followed by a lack of attention to the blood condition in many minor conditions of ill-health, and Dr. Fuller's book is a plea for more regular routine laboratory examination of the blood, and for wider and more efficient methods of treatment.

**The Diagnosis and Treatment of the Infectious Diseases.** By FREDERICK H. THOMSON, M.B., C.M. (Aberdeen), D.P.H. Pp. vi., 208. London : H. K. Lewis & Co. Ltd. 1924. Price 7s. 6d.—This book is intended for practitioners and attempts in two hundred pages to give a complete differential diagnosis and treatment of the thirteen common infectious diseases of this country. The book suffers in places from over-concentration of its contents, as such a volume, to be of value to practitioners for reference, should at least mention the rarer possible alternative diagnoses. For example, no mention is made of such a disease as malaria in the diagnosis of typhoid, a disease which in a sea-faring population is by no means rare and may resemble typhoid very closely. The description of fulminant diphtheria is hardly happy. To describe the soft, painless œdema of the neck which accompanies the disease as "cellulitis" is to convey an entirely wrong impression, for the swelling in diphtheria is due to effusion into the tissues, and is not a true inflammation. The omission of the Weil-Felix reaction as a serological test of typhus is unexpected. No mention is made under "Small Pox" of the present mild type nor of its peculiar diagnostic difficulties in some cases. In spite of these omissions, the book as a whole shows evidence of the wide personal experience of the author, and can be recommended to those who prefer, when in doubt, to consult a handy reference work rather than a ponderous treatise.

**The Nervous Patient.** By Millais Culpin, M.D. (Lond.), F.R.C.S. (Eng). Pp. viii., 305. London: H. K. Lewis & Co. 1924. Price 10s. 6d.—This book is a valuable addition to psychotherapeutic literature. It is essentially practical and intended for the general reader in medicine and surgery. The early part of the book summarises well-established psychological teaching in relation to the development of neuroses of all kinds. The author quickly proceeds to apply these principles to a wide field of medicine, surgery and gynæcology. All these chapters are worthy of close attention. Particularly interesting is the chapter on head injuries. There is one chapter, contributed by Mr. Inman, on ocular symptoms, headaches, squint and nystagmus which will be read with considerable interest both by general practitioners and ophthalmologists. Dr. Stanford Read contributes an interesting chapter on the major psychoses.

**Movement in Organic Disease.** By Ernest Kingscote, M.B., C.M. Edin. Pp. xix., 187. London: Baillière, Tindall & Cox. 1923. Price 10s. 6d. net.—The writer of this book, having had exceptional experience in the treatment of chronic affections of the heart and lungs, describes certain methods of treatment by special exercises by means of which he has secured most encouraging results even in cases of serious organic disease. These methods, and the indications for their employment, are described in such a way that any practitioner should be able to put them into practice in suitable cases; and there can be little doubt that cases frequently come under the care of every practitioner in which the application of these teachings would result in a degree of improvement which it would be difficult or impossible to achieve without having recourse to these forms of physical treatment. In the opening chapters the author draws attention to the fundamental importance of "normal breathing," meaning thereby a type of respiratory movement characterised by a greater excursion of the chest at the level of the nipples than at the level of the bases of the lungs. Where this type of breathing is present it proves that the mobility of the ribs and the elasticity of the lungs are fully operative, and that as a result of this the normal negative pressure in the chest, which is of such assistance to the venous circulation, is well maintained. The converse of this condition is, however, very frequently met with in persons living sedentary lives, and in the majority of sufferers from chronic affections of the lungs and heart. The writer makes it very clear what serious results are likely to follow upon such a condition of affairs and he shows how this condition can be easily recognised. Following this is a short but most important section in which the author describes how these cases of "fixed chest" can be treated by special breathing exercises which he has devised. The photographs reproduced in this section make it quite clear how the exercises should be performed. One of the most striking applications of these exercises is that in which they are applied in the treatment of emphysema. Other conditions in which these exercises in modified form are said to be of value are dilatation and fatty degeneration of the heart and angina pectoris. It is claimed that the results of Nauheim treatment are much more lasting if the "fixed chest" is mobilised by the Kingscote exercises. In conclusion, it may be stated that whilst this book contains a good many pages of somewhat questionable theory, it is well worth careful study for the sake of items of practical value.

**Clinical Memoranda for General Practitioners.** By ALEX THEODORE BRAND, M.D., C.M., V.D., and JOHN ROBERT KEITH, M.A., M.D., C.M. Second Edition. Pp. xiv., 375. London: Baillière, Tindall & Cox. 1923. Price 7s. 6d.—This is a general practitioner's scrap-book, culled apparently from a careful reading of the journals and supplemented by references to case books. In 350 pages it deals with a great variety of topics in medicine, surgery and obstetrics in a practical but scrappy manner. A busy practitioner will find here many useful "tips," but there is always a danger lest the perusal of books such as this should replace the careful study of more complete and systematic works.

**Internal Derangements of the Knee Joint.** By A. G. TIMBRELL FISHER, F.R.C.S. (Eng.). Pp. xii., 144. London: H. K. Lewis & Co. Ltd. 1924. Price 12s. 6d.—This book is the outcome of several years of careful and patient research by the author, chiefly at the Royal College of Surgeons, on the subject of the anatomy of internal derangements of the knee. It is introduced by a preface from the pen of Sir Arthur Keith. After a short introductory chapter dealing with the history of the subject, the rest of the matter is divided into a consideration of the semi-lunar cartilages, loose bodies, osteo-arthritis and certain other forms of internal derangement. The section on anatomy and physiology is exceedingly good, and it is very well illustrated. The same commendation applies to the description of the various lesions to which the semilunar cartilages are liable. In the operative treatment some stress is laid upon the advantages of a special method of exposure, which consists in a lateral displacement of the patella. Whatever the merits of this method may be, we doubt whether it is an original proposal, as an essentially similar method has been described by German orthopedists for some time. The section on loose bodies and also arthritis represent the author's original work on which he recently delivered the Hunterian Lecture. In these, as in all other sections of the book, a great deal of useful fact and suggestive theory is put forward, and the book as a whole is a most useful and practical summary of the matter with which it deals.

**Landmarks and Surface Markings of the Human Body.** By L. Bathe Rawling, M.B., B.C. (Cantab.). Sixth Edition. Pp. viii., 97. London: H. K. Lewis & Co. Ltd. Price 7s. 6d. net.—That this small book is firmly established in the estimation of those for whom it is intended is well shown by the fact that this is the sixth edition, and the thirteenth re-issue, in the course of ten years. There are few changes to record in the issue under notice, the text being left much as it was in previous editions. The illustrations are new and clear and generally accurate, although the amount of trachea which appears in the neck looks rather exaggerated in one or two of them. To those who are unacquainted with the book, the new edition may be confidently recommended as a concise and accurate guide to the study of surface anatomy.

**The Extra Pharmacopœia of Martindale and Westcott.** Eighteenth Edition. In two Volumes. Volume I. Pp. xxxviii., 1,163. London: H. K. Lewis & Co. Ltd. 1924. Price 27s. 6d.—The "Extra Pharmacopœia" of Martindale and Westcott has been so long the necessary companion of medical men and pharmacists that any review of a new edition must necessarily be a review of the new features only. The eighteenth edition is well up to the standard of its predecessors in providing the key to those methods of treatment of the most interest at the moment. The Dangerous Drugs Act, with its multiplicity of detail, is without doubt the cause of the greatest annoyance to both prescribers and dispensers. "Martindale" devotes eighteen pages to a survey of the Act and its amendments, giving explicit information of the procedure to be adopted by doctor, dentist, chemist, and wholesaler, a series of useful tables indicating the amount of the "Dangerous Drugs" in various preparations necessary to bring them under the Act, and a series of typical prescriptions, the perusal of which should effectively reduce the frequency of "referred back" prescriptions. The increasing use of intravenous injections is responsible for the addition of a table of intravenous doses of the drugs most often prescribed in this way, the table giving the actual dose and also the volume of diluent or solvent suitable in each case. Following the monograph on the pancreas and its preparations is a full account of Insulin, including methods of extraction, technique of injection, and dosage, followed by a very complete list of references to articles in the current literature on insulin. The much advertised class of colloids receives considerable attention, methods of preparation, both chemical and electrical and a detailed account of colloid therapy forming the subject of a lengthy chapter. Under the heading of "Alcohols" will be found mention of the present form of methylated spirit, only introduced in May of this year, and which has already proved a fertile source of trouble when used as a basis of skin lotions. In this connection it is to be noted by prescribers that the authors state authoritatively (p. 129) that "it is *illegal* to dispense *industrial* methylated spirit even on a doctor's prescription, *e.g.* for a cooling skin lotion." A section of about eighty pages is devoted to vaccines and antitoxins, commencing with an account of the theory of immunity and concluding with a survey of sensitised vaccines. A very complete index is provided, without which the utility of a book of this kind is much lessened.

**An Introduction to the Histology of Tumours.** By HAROLD A. HAIG, M.B., B.S., D.P.H. Pp. vii., 128. London: Henry Kingston. 1924. Price 7s. 6d. net.—The object of this small book of 124 pages is "to aid students and working pathologists in the diagnosis of tumours by the microscope," a formidable and responsible task, requiring greater clarity of exposition and a larger share of the gifts of judicial selection and typical illustration than we find in this book. An introduction plunges the reader into the minutiæ of high-power histology, leaving him too ready to believe that malignancy can be diagnosed from the characters of individual cells. A conventional description of the commoner tumours follows, with a large number of photographs which we cannot highly commend. Working pathologists need more detail, and students demand greater simplicity, than this book affords.

**Text-Book of Pathology.** By ROBERT MUIR, M.A., M.D., Sc.D., F.R.S. Pp. vii., 774. London: Edward Arnold & Co. 1924. Price 35s.—Professor Muir's book should prove of great value to those who wish to keep abreast with modern pathology, as well as to the medical student for whom it is primarily intended. It deals essentially with the structural changes in disease, but where necessary for a clearer understanding of the subject the author discusses briefly the results of chemical and experimental research. Systematic bacteriology and diseases of the special senses and of the skin have been omitted, and only the commoner tropical diseases likely to be met with in this country are described. A short account of the more important animal parasites is given, and also of the chief congenital abnormalities. The student will find no difficulty in following the author's description of the various pathological processes, nor will his interest fail to be stimulated by the constant reference to the functional disturbances caused by them. The illustrations are excellent. The book will certainly become one of the most popular student's text-books on the subject.

**The Journal of Cancer.** No. 1, January, 1924. Dublin: Alex. Thom. & Co. Ltd. Price 2s. 6d. quarterly.—A new Journal professing to be "the only magazine devoted to cancer research," and "designed as a record of the study, experiments and general practice in the treatment of cancer." This, the first number, contains a short and unduly optimistic editorial, several papers on deep X-ray therapy and an article in smaller print entitled "The Cancer Problem," by Dr. W. M. Crofton. There are several misprints in an article by the Editor. The author of the first article on deep X-ray therapy acknowledges his results to be premature. The second and third articles dealing with the same subject are rather alarming. The first describes many contra-indications to, and serious complications of the treatment; the second emphasises the difficulty in finding a safe dosage. Dr. Crofton's article is speculative and discursive as to cancer and surprisingly confident as to its successful treatment by vaccines.

**A Manual of Diseases of the Eye.** By Charles H. May, M.D., New York, and Claude Worth, F.R.C.S., Eng. Fifth Edition. Pp. viii., 460. Baillière, Tindall & Cox. 1924. Price 15s.—The new edition of this work is, like its predecessors, written essentially for the medical student and general practitioner. It has now been brought well up to date. The coloured plates, which have always been such a feature of this book, still retain their excellence, and in themselves constitute a very useful atlas of the diseases of the eye. The chapters on refraction and the ordering of glasses are simply and concisely written, and should enable anyone to obtain a clear insight into this often rather difficult section of ophthalmology. The chapter on ocular therapeutics cannot but be of considerable practical use, giving as it does many useful prescriptions and the various methods of applying them to the eye. Taken as a whole, the book can be confidently recommended to the student who requires a sound practical but short work on ophthalmology, and to the general practitioner who is in need of a treatise on the subject dealing with those diseases which he is most likely to meet in his daily work.

**Clinical and Operative Gynæcology.** By J. M. MUNRO KERR. Pp. xiv., 832. London: H. Frowde & Hodder & Stoughton. 1922. Price 50s. net.—This book is a very welcome addition to the numerous works on this subject. Its value is enhanced by the fact that the author presents a record of his own clinical experience and an exposition of his own technique. The book is therefore free from what is, in our opinion, a too prevalent fault, namely the inclusion of long accounts taken from other writers of their opinions without giving the data on which those opinions were formed. It is unnecessary to state that those engaged in teaching should be especially careful to avoid this fault, as more than any other it leads to the perpetuation of opinions, the errors of which, and the inaccurate deductions which lead up to them, are liable to be carried forward in an intensified form by uncritical readers. For example the author's experience is that the proportion of cases of salpingitis is not, as often alleged, due in the majority of cases to gonorrheal infection and in the minority to infection following pregnancy, but the reverse. Of his own cases 60 per cent. were due to infection following pregnancy and 40 per cent. to gonorrhœa, which accords with our own experience. The other statement is to the effect that chorio-epithelioma is a common sequela of hydated mole, instead of what is the fact, that in a large proportion of cases of chorio-epithelioma hydatid mole has been a precursor. The relative frequency of the two conditions is sufficient evidence of the untruth of the former statement. The general arrangement of the work is much to be commended, it avoids unnecessary detail, while giving all the information that the clinician will find necessary. The clinical presentation of cases in which difficulties of diagnosis occurred is admirable, and not least so in those cases in which mistakes in diagnosis had been made by the author himself. The chapter on medical as opposed to operative treatment and the author's remarks on the limitations of the latter will be found most interesting and valuable, and may serve as a corrective to those who seem to consider that operative treatment is the one method of dealing with gynæcological affections. The author wisely insists on the correct appreciation of the anatomical facts on which operative procedure should be based, and, in his presentation of biological factors, on the importance of a correct reading of facts furnished by the biochemist and physiologist. The necessity for basing gynæcological methods on the ancillary pure sciences has been in the past overlooked, chiefly owing to the difficulties inherent in the endeavour to acquire the experience necessary for the practice of the handicraft. Operative technique is dealt with in the last section of the work by means of a comprehensive series of plates with lucid explanations attached. We can strongly recommend this work as a guide to those who, having attained a certain stage of experience in the subject, wish to extend it by comparison with that of others. It is true that many of the present-day procedures and fashions are not dealt with in detail, but those who wish to study a work based on common sense and the application of scientific knowledge will find it not only valuable, but most interesting and easy reading, and a wholesome corrective to some of the *ex cathedrâ* statements found in too many of the other manuals.

**Handbook of Skin Diseases.** By FREDERICK GARDINER, M.D., B.Sc. Second Edition. Pp. 248. Edinburgh: E. & S. Livingstone. 1924. Price 10s. 6d.—A successful text-book demands subsequent editions. That Gardiner's handbook has met with success is, partly, because in a very small compass and at moderate cost it provides students with some idea of diagnosis and treatment in the commoner diseases of the skin. There are very few dermatological handbooks which a student can carry in his pocket and have ready for reference in the out-patient room. In the present issue additions have been made to the letterpress, and coloured plates have been added, which have improved the book; the paper also permits of better reproduction of the photographs, but it is to be hoped that in subsequent editions no further increase in bulk will occur, or the usefulness as a pocket handbook will largely be lost. The price also has increased from 6s. to 10s. 6d., which is of moment to students. There are important omissions and careless mistakes in the wording, but the book serves a useful purpose, and can be recommended to students till they can afford a larger and more complete text-book.

**Handbook of Sanitary Law.** By B. BURNETT HAM, M.D., D.P.H. Edited by HENRY R. KENWOOD, C.M.G., M.B., F.R.S. (Edin.), D.P.H. Ninth Edition. London: H. K. Lewis & Co. Ltd. 1923. Price 5s. net.—This little book presents in a convenient form extracts from all the Acts dealing with Sanitary Law which require memorisation by the D.P.H. student. The ninth edition brings the subject matter up to date by the addition of all recent enactments. It will doubtless retain its popularity with students as the best book from which to absorb its arid subject.

